# Pentixafor PET/CT for imaging of chemokine receptor 4 expression in esophageal cancer – a first clinical approach

**DOI:** 10.1186/s40644-021-00391-w

**Published:** 2021-02-12

**Authors:** Philipp Linde, Christian Baues, Simone Wegen, Maike Trommer, Alexander Quaas, Johannes Rosenbrock, Eren Celik, Simone Marnitz, Christiane J. Bruns, Thomas Fischer, Klaus Schomaecker, Hans-Juergen Wester, Alexander Drzezga, Lutz van Heek, Carsten Kobe

**Affiliations:** 1 Department of Radiation Oncology, University Hospital of Cologne, University of Cologne, Kerpener St 62, 50937 Cologne, Germany; 2 Department of Pathology, University Hospital of Cologne, University of Cologne, Cologne, Germany; 3 Department of General, Visceral, Tumor and Transplantation Surgery, University Hospital of Cologne, University of Cologne, Cologne, Germany; 4 Department of Nuclear Medicine, University Hospital of Cologne, University of Cologne, Cologne, Germany; 5 Department of Radiochemistry, Technische Universität München, Garching, Germany

**Keywords:** CXCR4, Pentixafor, Esophageal cancer, Molecular imaging, PET/CT, Radiotherapy

## Abstract

**Background:**

Expression of CXCR4, a chemokine (C-X-C motif) receptor that plays a central role in tumor growth and metastasis of circulating tumor cells, has been described in a variety of solid tumors. A high expression of CXCR4 has a prognostic significance with regard to overall and progression-free survival and offers a starting point for targeted therapies. In this context, [68]Ga-Pentixafor-Positron Emission Tomography/Computer Tomography (PET/CT) offers promising possibility of imaging the CXCR4 expression profile. We set out to compare a [18F] fluorodeoxyglucose (FDG)-PET/CT and a [68Ga]Pentixafor-PET/CT in (re-)staging and radiation planning of patients with localized esophageal cancer.

**Materials and methods:**

In this retrospective analysis, ten patients, with adeno- or squamous cell carcinoma of the esophagus (*n* = 3 and *n* = 7, respectively), which were scheduled for radio (chemo) therapy, were imaged using both Pentixafor and FDG PET/CT examinations. All lesions were visually rated as Pentixafor and FDG positive or negative. For both tracers, SUVmax was measured all lesions and compared to background. Additionally, immunohistochemistry of CXCR4 was obtained in patients undergoing surgery.

**Results:**

FDG-positive tumor-suspicious lesions were detected in all patients and a total of 26 lesions were counted. The lesion-based analysis brought equal status in 14 lesions which were positive for both tracers while five lesions were FDG positive and Pentixafor negative and seven lesions were FDG negative, but Pentixafor positive. Histopathologic correlation was available in seven patients. The CXCR4 expression of four non-pretreated tumour lesion samples was confirmed immunohistochemically.

**Conclusion:**

Our data shows that additional PET/CT imaging with Pentixafor for imaging the CXCR4 chemokine receptor is feasible but heterogeneous in both newly diagnosed and pretreated recurrent esophageal cancer. In addition, the Pentixafor PET/CT may serve as complementary tool for radiation field expansion in radiooncology.

**Supplementary Information:**

The online version contains supplementary material available at 10.1186/s40644-021-00391-w.

## Introduction

In 2018, there were 572,034 new diagnoses of esophageal cancer and 508,585 deaths caused by the disease worldwide [1]. Malignancies of the esophagus cause about 3.5% of all cancer deaths in men and 1.2% in women in Germany [2]. Histopathologically, esophageal carcinoma usually manifests as adenocarcinoma or squamous cell carcinoma. Curative treatment with operation alone in T1–2a tumors and tri-modal treatment with radio-chemotherapy and surgery in locally advanced stages is restricted to patients with no distant metastasis. Since most patients present with advanced disease given late onset of symptoms, they will be directed to non-surgical treatment such as radio- and/or chemotherapy, leading to an expected 5-year survival of less than 15% [3]. As a result, new treatment options including personalized medicine targeting specific molecular markers, are urgently needed.

Positron emission tomography–computed tomography (PET/CT) using fluorodeoxyglucose (FDG) has become an established tool for staging patients with esophageal cancer, since it has the highest sensitivity for the evaluation of distant metastasis [4]. Furthermore FDG PET/CT is used in esophageal cancer to predict response to chemotherapy at an early stage of treatment. Here, FDG PET/CT has shown its predictive value concerning treatment outcome [5].

Chemokine (C-X-C motif) receptor 4 (CXCR4) and its ligand, the alpha-chemokine CXCL12, has been described to play a central role in tumor growth and progression, tumor invasiveness and metastasis [6]. Overexpression of this receptor has been reported in more than 30 different types of cancer [7–10]. Importantly, the CXCL12/CXCR4 axis has been identified as a target for drugs in human tumors due to its critical role in promoting and maintaining cancer stem cells [11]. The CXCL12/CXCR4 axis mobilizes heterogeneous signaling pathways that foster adhesion, chemotaxis, migration, cell proliferation and survival [12].

In primary esophageal cancer, overexpression of CXCR4 was found to be associated with clinicopathological features e.g. gender, histological differentiation, tumor depth, and status of lymph node metastasis, and poor prognosis [13]. Probably CXCR4 expression can be expected in more than 50% of esophageal cancer patients [14]. Wang et al. could show that the ability of esophageal cancer stem cells to spread and metastasize through ERK1/2 signaling pathway could be inhibited by blockage of CXCR4 with inhibitors or shRNA approaches both in vivo and in vitro studies [15]. Zhang et al. suggest that miR-302b, a small non-coding RNA, may be a novel cancer-related inflammation (CRI) regulating miRNA [16]. It inhibits CRI critical pathway and downstream cytokines expression through targeting CXCR4 amongst others, resulting in decrease of tumor growth.

In 2011, a radiolabelled CXCR4-ligand ([^68^Ga] Pentixafor) for PET imaging has been developed promising diagnostic improvement and targeted treatment [17–19]. Herrmann et al. showed first results of CXCR4-targeted radiotherapy with Lu-marked CXCR4 specific agent pentixather [20].

We hypothesize that additional PET/CT imaging with Pentixafor to visualize the chemokine receptor CXCR4 is feasible in both newly diagnosed and pretreated recurrent esophageal cancer and gives complementary information to FDG PET/CT. Radiolabeled chemokine ligands could contribute as additive imaging for in vivo identification and non-invasive tumor characterization. In this context, they could extend the staging information and possibly enable better patient stratification.

The aim of this analysis is to report on a direct comparison Pentixafor PET/CT and FDG PET/CT in patients with esophageal cancer in (primary) staging as well as part of planning PET CT prior to radio-chemotherapy as a feasible option, as in order to evaluate further treatment options for selected patients.

## Materials and methods

### Patients

Both FDG and Pentixafor PET/CT were performed for clinical use. In this retrospective analysis we included ten adult esophageal cancer patients suffering of advanced or relapsed disease, and in whom radio (chemo) therapy was planned. We restricted our analyses to PET/CT scans which were performed between November 2014 and March 2015. All consecutive patients underwent an additional Pentixafor PET/CT within a mean period of 8 days (range 2–35) in order to further characterize their CXCR4 expression and to evaluate potential CXCR4-related treatment options.

### PET/CT imaging protocol

All PET/CT examinations were performed on a Biograph mCT Flow – Edge 128 PET/CT system (Siemens Medical Solutions) with a 128-slice spiral CT component from the base of the skull to the mid-thigh after patients had fasted for 6 hours. The CT scan for the attenuation correction was performed as a native non-diagnostic scan with a tube current of 30 mAs and a maximum voltage power of 120 kVp. The CT scan was followed by a PET emission scan. FDG was synthesized in house as previously described [21]. Injection activities for FDG were the following: mean 299.2 MBq (range 242–394 MBq). Uptake time between injection and scan was mean 01:11 min (range 00:57–01:37 min). To meet the criteria for European Association of Nuclear Medicine and its Research Ltd. (EARL) certification, reconstruction was performed via ordered subset expectation maximization (OSEM) algorithm (four iterations and twelve subsets), followed by an intrinsic 5 mm Gaussian filter in all directions. Pentixafor was prepared in a fully automated procedure using a module equipped with a single-use cassette kit (Scintomics GmbH, Germany; ABX, Germany) [12]. For radiolabelling, 25 μg of the precursor CPCR4.2 (ABX, Germany) were used. Per patient, approximately 8.3 μg of the [68]Ga-Pentixafor was then applied. Injection activities for Pentixafor were the following: mean 174.5 MBq (range 129–226 MBq). Uptake time between injection and scan was mean 01:04 min (range 00:59–01:11 min).

All patients underwent the PET/CT examinations as part of the clinical workup in order to potentially optimize their individual treatment and with diagnostic intent. All patients signed informed consent in regard of the scientific evaluation of their data. The retrospective evaluation of the data was approved by our ethics committee and conformed to the provisions of the Helsinki Declaration.

### Image analysis

PET images were independently analyzed with reference to the contrast CT images by two experienced nuclear medicine specialists. All differences of opinion in interpretation were resolved by consensus. For all image analyses OSEM reconstruction was used. For visual analysis, uptake of all lesions (reference regions) was assessed for both FDG and Pentixafor as positive or negative taking the mediastinal blood pool (MBP) as a reference region. A lesion was considered Pentixafor- or FDG-positive when measured ≥ MBP. In addition, we also considered the liver as reference [22]. Tumor to background ratios were calculated as SUVmax of tumor divided by SUVmean of background (Table 2). For quantitative evaluation the maximal standardized uptake value (SUVmax) was measured in all the lesions, too. The mean standardized uptake value (SUVmean) in the reference regions were determined by placing a sphere with a diameter of 2 cm in the upper right part of the liver, in the mediastinum (refers to ascending aorta), in the spleen, in the bone marrow and in the brain.

### Immunohistochemistry

The reference pathology was performed by one experienced pathologist; again, uncertainties in interpretation were resolved by consensus in cooperation with another pathological specialist with extensive expertise. To exclude a possible influence of concomitant therapy on receptor surface expression from the beginning, biopsies (oesophagogastroduodenoscopy (OGD), *n* = 4) were performed shortly after PET imaging and before starting treatment. Immunohistochemistry (IHC) was performed on formalin-fixed and paraffin embedded material using the rabbit monoclonal CXCR4 antibody from Cell Signaling Technology (clone D4Z7W; order code 97680; EDTA-buffer, dilution 1:800) on the automated Leica Bond stainer. The CXCR4 expression, which means CXCR4-positive inflammatory cells in the tissue, will be given in a scale range from 0 to 4 (0 = no CXCR4 (0–3 CXCR4 positive cells/high power field (HPF); 1 = low CXCR4 (4–10 CXCR4 positive cells/HPF); 2 = intermediate (11–25 CXCR4 positive cells/HPF); 3 = strong (> 26 CXCR4 positive cells/ HPF); 4 = not available).

### Statistics

Quantitative measurements are presented using descriptive statistics. SUVs in the lesions, background regions and their ratios were compared using Wilcoxon matched-pair signed-rank (2 samples) test. Statistical analyses were performed using IBM SPSS statistics (version 22, IBM SPSS Statistics, Armonk, NY, USA) and R Core team (2017, version 4.0.3, Vienna, Austria).

## Results

### Clinical findings

Eight patients presented with advanced stage disease (infiltration of the tunica adventitia or neighboring structures (T3–4 stage)), two subjects with local disease (infiltration into the muscularis propria layer (T2 stage). Two patients underwent Pentixafor PET/CT as part of restaging after radiotherapy (#8) or radiochemotherapy (#9); the therapies were completed 11 and 9 months ago. Patient #4 was treated with platinum-containing postoperative radiochemotherapy for first-line laryngeal cancer 24 months prior to diagnosis of esophagus cancer. In the majority of cases there was no intervention (e.g. chemotherapy, surgery) done between the scans; however, patient #6 received three fractions, patient #7 one fraction of a radiotherapy in 1.8 Gray single dose before Pentixafor PET/CT (fractions of radiotherapy is equivalent to days before PET/CT). In patients #1,4,6 and 7, CXCR4 expression was determined in cell sample obtained by OGD. In the remaining patients either no cell material was present (*n* = 3) or the expression was determined after previous radiotherapy ± chemotherapy (*n* = 4).

**Fig. 1:**
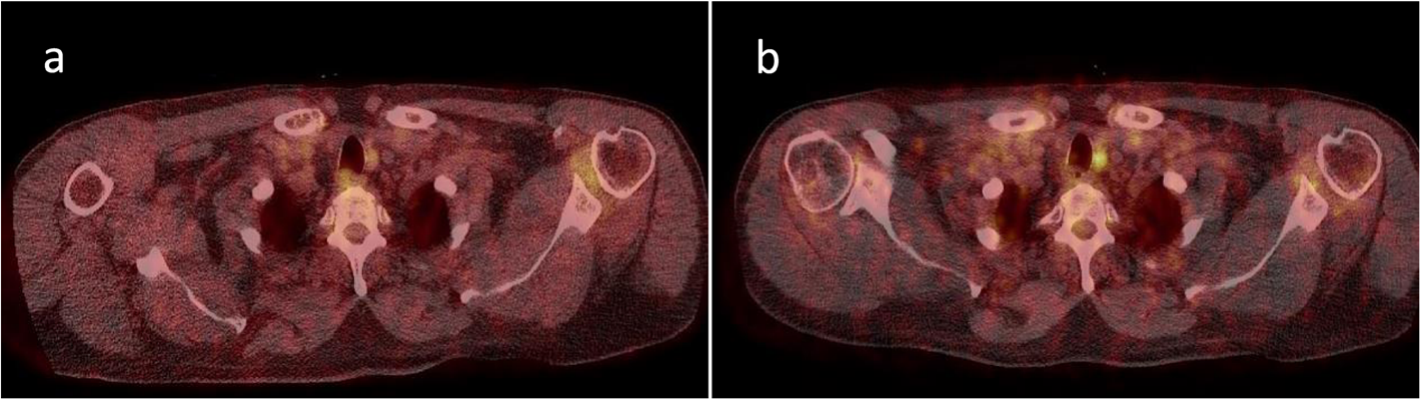
Esophageal carcinoma patient (#1) with (**a**) positive FDG-uptake and a suspicious lesion enhanced by Pentixafor PET/CT (**b**). Arrow indicates the lesion

**Fig. 2:**
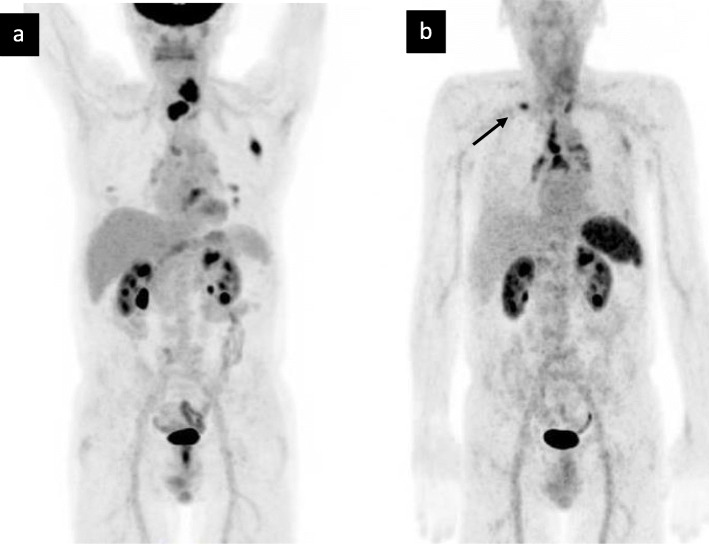
Key lesion rather peripher located to the primary tumor. Disconcordance of (**a**) FDG PET/CT and (**b**) non-invasive Pentixafor PET/CT imaging in one patient suffering from esophageal cancer. The lesion demonstrates high CXCR4 expression; local response was able to be shown in re-staging PET/CT after radiochemotherapy. Arrow indicates the lesion

**Fig. 3:**
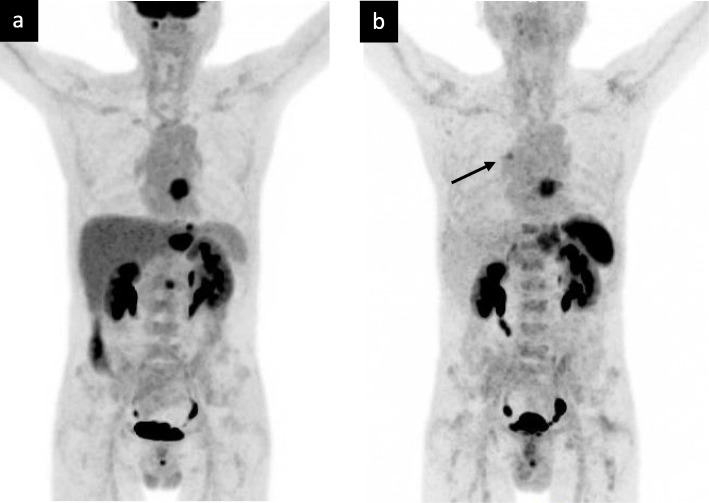
Key lesion with close relation to the primary tumor: Esophageal carcinoma patient (#4) with (**a**) negative FDG-uptake and an additional suspicious lesion hilary right enhanced by Pentixafor PET/CT (**b**). The abdominal uptake is caused by an inserted gastric feeding tube. Arrow indicates the hilary lesion

Detailed patient characteristics (eight males, mean age 67, standard deviation 8, range 53–76 years) are given in Table 1. In order to optimise the radio-oncological treatment of all patients, the suspicious lesions resulting from the additional information obtained with the Pentixafor PET/CT (compare Figs. 1 and 2) were included in clinical staging. An oncological upstaging was defined for patient #10 in consensus. In four patients (#1, #4, #9, #10) we found additional lesions in Pentixafor PET/CT as compared to FDG PET/CT. We included lesions in the irradiation volume if we found them Pentixafor PET-positive. By definition, most lesions were already located in the area to be irradiated (e.g. lymph drainage area). The additional lesion in the left rib at #10 did not lead to an expansion of the irradiation field. A follow-up was intended here. Consequently, the irradiation field was adjusted based on the gain in information from the Pentixafor PET/CT in one patient (#4, lesion near the right hilus; FDG negative, Pentixafor positive; Fig. 3).

**Table 1:** Demographic and clinical characteristics of participants

Patient no.	Age	Gender	Grading	UICC TNM	Sample origin	Histology
#1	61	m	GX	cT2 cN1 cM0	I	SCC
#2	72	m	G2	cT4 cN2 cM0	III	SCC
#3	76	m	G3	cT2 cN1 cM0	II	Adeno
#4	58	m	G3	uT3 cN2 cM0	I	SCC
#5	70	m	G2	uT3 cN0 cM0	II	Adeno
#6	75	m	G2	uT3 cN+ cM0	I	Adeno
#7	53	m	G2	cT3–4 cN2 cM0	I	SCC
#8	74	w	GX	uT3 cN+ cM0	III	SCC
#9	70	w	GX	uT3 cN1 cM0	II	SCC
#10	61	m	G2	cT3–4 cN2 cM1	III	SCC

*Adeno* Adenocarcinoma, *SCC* Squamous cell cancer, *UICC TNM* The Union for International Cancer Control Tumor Node Metastasis Classification of Malignant Tumours, 7th Edition, *I* collection by oesophago-gastroduodenoscopy, *II* collection by surgery, *III* no sample available for CXCR4 expression determination

### SUVs in background for FDG and Pentixafor

Mean SUVmax in the tumor lesions were 6.9 ± 4.6 for FDG and 4.7 ± 2.5 for Pentixafor respectively. The mean SUVmean in the reference regions for FDG and Pentixafor were 2.5 ± 0.4/1.4 ± 0.3 in the liver, 1.7 ± 0.5/1.7 ± 0.4 in the mediastinum, 1.8 ± 0.3/5.6 ± 1.0 in the spleen, 1.3 ± 0.5/1.6 ± 0.8 in the bone marrow and 7.7 ± 2.0/0.2 ± 0.1 in the brain (supplementary Table 1). As we suspected, the results from supplementary Table 1 show that FDG and CXCR4 have different biodistributions. We have recognized the SUV on Pentixafor as feasible; however, the SUV must be treated with caution as it is not validated or standardized yet.

### Visual analysis

A total of 26 lesions were counted. Patient-based analysis FDG and Pentixafor revealed comparable results in 4/10 patients. Lesion-based analysis showed equal results in 14 lesions which were positive for both tracers while 5 lesions were FDG positive and Pentixafor negative and 7 lesions were FDG negative but Pentixafor positive (Table 2).

**Table 2:** Intraindividual comparison of lesion uptake FDG vs. Pentixafor

Patient no.	Localization of the lesion	Quantitative analyses		Visual analyses		Adeno/SCC	CXCR4 expression^b^
		FDG SUVmax	Pentixafor SUVmax	FDG^a^	Pentixafor^a^		
**#1**	paratracheal left	2.2	5.2	0	2	SCC	1
**#2**	mediastinal	10.1	3.2	2	0	SCC	4
	infraclavicular	3.4	4.5	2	1		
**#3**	hip	7.1	2.7	2	2	Adeno	1
	thigh	10.5	3.9	2	2		
	adrenal gland left	3.3	2.9	2	2		
	left paraaortal lymph node	3.3	1.3	1	0		
**#4**	esophagus	7.3	5.9	2	2	SCC	3
	coeliacal	9,0	5,2	2	2		
	axillary right	0,85	1,73	0	2		
	hilary right	2,0	3,5	0	2		
	gastric curvature	5,6	4,1	2	2		
**#5**	esophagus	6.6	6.5	2	2	Adeno	0
**#6**	mediastinal	14.4	4.2	2	0	Adeno	2
**#7**	mediastinal	15.8	4.8	2	2	SCC	3
**#8**	adrenal gland left	2.5	5.0	2	1	SCC	4
	esophagus	3.3	3.9	1	0		
	lung upper lobe	4.9	1.5	2	0		
**#9**	adrenal gland left	2.7	12.0	0	2	SCC	2
**#10**	infracarinal	2.7	9.1	0	2	SCC	4
	mediastinal	4.1	9.8	1	2		
	supra right	2.2	5.4	0	2		
	supra left	2.8	6.6	0	2		
	esophagus	14.4	3.7	2	1		
	rib left	9.2	3.8	2	1		
	cervical left	13.7	4.1	2	1		
**SUM**	26						

*Adeno* Adenocarcinoma, *MPB* Mediastinal blood pool, *SCC* Squamous cell cancer

^a ^0= < MBP, 1 ≥ MBP, 2 ≤ Liver

^b^ 0 = no CXCR4; 1 = low CXCR4; 2 = intermediate; 3 = strong; 4 = not available

### Quantitative analysis

Mean intensity of uptake in the lesion was higher, but not statistically significant, for FDG, which is reflected by the mean SUVmax in the lesions 6.9 for FDG and 4.2 for Pentixafor (*p* = 0.075). For mean SUVmean in the reference regions for FDG and Pentixafor were 2.5 and 1.4 in the liver (*p* < 0.001), 1.7 and 1.7 in the mediastinum (*p* = 0.635), 1.8 and 5.6 in the spleen (*p* < 0.001), 1.3 and 1.6 in the bone marrow (*p* = 0.4316), and 6.1 and 0.2 in the brain (*p* = 0.0039), reflecting the higher physiological uptake of FDG in the brain and the liver, and the higher affinity of Pentixafor to the spleen. When choosing the liver as reference region the mean ratio of the SUVmax in the lesion to the SUVmean within the liver was 3.0 for FDG and 3.8 for Pentixafor (*p* = 0.25) (Table 3).

**Table 3:** Tumor to background ratio (TBR) liver/mediastinum for FDG and Pentixafor

Patient no.	Localization of the lesion	FDG		Pentixafor	
		TBR liver	TBR MBP	TBR liver	TBR MBP
**#1**	paratracheal left	1,0	1,3	2,6	3,5
**#2**	mediastinal	5,1	6,7	2,9	2,1
	infraclavicular	1,7	2,3	4,1	3,0
**#3**	hip	2,2	3,4	2,5	1,1
	thigh	3,2	5,0	3,5	1,6
	adrenal gland left	1,4	2,1	2,6	1,2
	left paraaortal lymph node	1,0	1,6	1,2	0,5
**#4**	esophagus	2,5	3,7	4,9	3,7
	coeliacal	3,1	4,5	4,3	3,25
	axillary right	0,3	0,4	1,4	1,1
	hilary right	0,7	1	2,9	2,2
	gastric curvature	1,9	2,8	3,4	2,6
**#5**	esophagus	2,8	4,1	4,1	3,4
**#6**	mediastinal	5,1	6,3	3,0	2,5
**#7**	mediastinal	6,6	22,6	4,0	5,3
**#8**	adrenal gland left	0,9	1,4	4,2	2,9
	esophagus	1,2	1,8	4,2	2,9
	lung upper lobe	1,8	2,7	1,3	0,9
**#9**	adrenal gland left	1,2	1,5	12,0	6,0
**#10**	infracarinal	1,2	1,9	5,4	4,8
	mediastinal	1,8	2,9	5,8	5,2
	supra right	1,0	1,6	3,2	2,8
	supra left	1,2	2,0	3,9	3,5
	esophagus	6,3	10,3	2,2	1,9
	rib left	4,0	6,6	2,2	2,0
	cervical left	6,0	9,8	2,4	2,2

*MPB* Mediastinal blood pool

### Immunohistochemistry

**Fig. 4:**
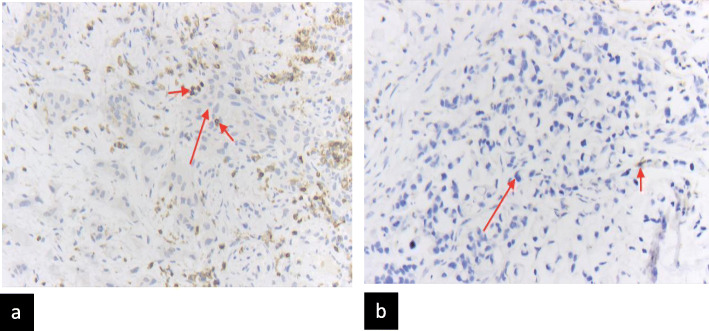
Immunohistochemistry: CXCR4 (both photos in same magnification: 200x) coloring in (**a**) squamous cell carcinoma: (long arrow) is strong positive for CXCR4 (CXCR4 positive inflammatory cells marked with short arrow); and (**b**) adenocarcinoma (with signet ring cell features; long arrow) scored as negative (less than three CXCR4 positive cells in high power field/HPF; one CXCR4 positive cell marked with short arrow)

We were able to make semi-quantitative statements about the quantity and number of CXCR4-positive inflammatory cells in the tissue. In 7/10 patients imaging results could be compared to immunohistological staining for CXCR4 derived from surgical specimens (*n* = 3) as well as biopsies from the primary (*n* = 4) (Table 1). Regarding the histological evaluation of CXCR4 expression, 2/10 samples (#1, #3) were rated “low” (4–10 CXCR4 positive cells/HPF), 2/10 “intermediate” (11–25 CXCR4 positive cells/HPF) (#6, #9) and 2/10 “strong” (> 26 CXCR4 positive cells/HPF) (#4, #7) positive. Patient #5 was scored negative (0–3 CXCR4 positive cells/HPF) and 3/10 samples (patients #2, #8 and #10) were not scored at all (compare Fig. 4).

## Discussion

Our observation of in vivo imaging of CXCR4 expression in humans with both newly diagnosed as well as pre-treated, recurrent esophageal cancer suggest that chemokine receptor 4 (CXCR4) expression in esophageal cancer is not an unusual condition and can be assessed non-invasively by PET/CT and the CXCR4-directed radiopharmaceutical [68Ga]Pentixafor. However, it should be highlighted that CXCR4 expression is not specific for esophageal cancer.

Nevertheless, for almost all tumor lesions we could not find any significant exceptions for high tumor background ratio compared to FDG PET/CT as a reference. This underlines that CXCR4 may prove to be a promising target for endoradiotherapy in selected cases, as recently proposed e.g. by Lapa et al. [23–25]. Recent reports evaluating biopsy samples of esophageal cancer tumors demonstrated a high intensity of CXCR4 receptor expression [26, 27]. Additionally, chemokine receptor expression was a predictor of poor recurrence-free and overall survival [28–31].

The preliminary results of this retrospective analysis indicate a heterogeneity of CXCR4 expression and imply that the information may be considered complementary to Computerized Tomography (CT), endoscopic ultrasound (EUS) and FDG PET/CT within three-modality staging [32–34]. In case of overexpression of CXCR4 in esophageal tumours or their metastases, an additional Pentixafor PET/CT could detect further localisations not visible in FDG PET/CT. As an example, Philipp-Abbrederis et al. demonstrated the benefits of Pentixafor PET imaging in a subset of multiple myeloma patients where specificity and contrast were superior to [18F]FDG [35]. Particularly in view of the low to moderate sensitivity of FDG-PET/CT for lymphonodal staging and the distinction between active tumor tissue and local esophagitis [36].

As Werner et al. noted, future attempts for possible applications of CXCR4-directed imaging could focus on the characterization of lesional heterogeneity by conducting dual radiotracer studies (in coexistence with [18F]FDG) to visualize different levels of tumor differentiation and predict metastases with prognostic relevance. For example, a CXCR4-directed PET/CT could help to visualize receptor-positive cancer stem cells that are considered to be in particular resistant to radiation or chemotherapy [37].

Detailed information of the exact tumor stage is essential for decision making in oncological treatment strategies. Since the treatment of esophageal cancer is a stadium-adapted therapy, accurate and correct staging is of particular importance. In the case of neoadjuvant radiotherapy, the size of the RT field varies depending on the lymph node involvement. Pentixafor PET/CT could have therapeutic impact, e.g. on the size of the field to be irradiated in patients with esophageal cancer. In addition, and of course, the detection of distant metastases would lead to a change in the therapeutic goal towards palliative treatment. Considering the small number of our cases, it appears that a high CXCR4 expression, e.g. samples #4 and #7, is accompanied by a higher T-stage of disease. However, it must be taken into account - which has already been critically demonstrated by Lapa et al. - that receptor presentation on the tumor cell surface appears to be highly dynamic and is influenced by a variety of factors, including previous therapy [38].

In the majority of studies, overexpression of CXCR4 had been investigated in esophageal tumors or its metastases. Łukaszewicz-Zając et al. examined the serum concentrations (SC) of chemokine CXCL12 in patients with esophageal cancer compared to a healthy control group. CXCL12 SC were significantly higher, as those of its receptor CXCR4. This suggests the possibility that CXCR4 could become a prognostic factor in a combined analysis with classical tumor markers such as carcinoembryonic antigen and C-reactive protein levels [39].

In addition, Koishi et al. were able to show that persistence of positive CXCR4 expression is implicated in tumor aggressiveness and poor prognosis in esophageal (squamous cell) cancer (ESCC) after neoadjuvant chemoradiotherapy (nCRT) [40]. Furthermore they showed that nCRT may improve the prognosis of ESCC via CXCL12-CXCR4 signaling pathway [41]. Sasaki et al. demonstrated that positive CXCL12 expression was closely related to tumor development [42]. We can therefore expect that patients, including those with advanced diseases, can be monitored by CXCL12 and thus the effectiveness of multimodal therapies can be observed. At this time, it remains unclear whether chemoradiotherapy could affect CXCL12-CXCR4 signaling or not, since the status of CXCR4 expression after chemoradiotherapy was not available in our cohort.

Nevertheless, our data suggests that a noticeable CXCR4-positive immune or epithelial cell population might accumulate in tumors and we have been able to demonstrate a major fraction of tumor cells to be CXCR4-positive. Our samples #3, #5 and #6 – all examined as adenocarcinoma, all got treated with radiochemotherapy protocol according to CROSS trial, CXCR4 expression was determined in the surgical specimen – ranged from none to intermediate expression, as well as the squamous cell cancer (SCC), which ranged up to strong expression (#4, #7) fixed in OGD sampling [40]. SCC sample #9 received CROSS protocol before determination in surgical specimen, too and was rated intermediate. Of course, it must be critically considered that CXCR4 expression can be up- or underregulated by chemo- and radiotherapy and should therefore be considered with caution [43–45].

Still, there are differences in regard of in vitro and in vivo distribution, when it comes to CXCR4 overexpression. Vag et al. observed that the reported in vitro evidence of CXCR4 overexpression in malignancies such as pancreatic cancer, non-small cell lung cancer, prostate cancer, melanoma, breast cancer, hepatocellular carcinoma, glioblastoma, and sarcoma does not depict the in vivo distribution revealed by Pentixafor PET/CT [46]. These results could potentially differ because 68Ga-Pentixafor PET binds to membrane-associated chemokine receptors. CXCR4 expression levels determined by either transcript or whole-cell protein level analysis is not necessarily representative of the CXCR4 expression level on the cell surface [47, 48]. Therefore, there could be a significant discrepancy between CXCR4 expression profiles determined by analysis of transcript or whole-cell protein protein level analysis and by in vivo quantification of CXCR4 using PET probes. Shim et al. demonstrated that CXCR4 expression in lymph node metastases in breast cancer originates mainly in the cytoplasm. Other relevant factors in this context could be the overexpression of CXCR4 on cancer stem cells, which are believed to represent a drug-resistant cell population.

Even after definitive treatment, esophageal cancer features a high risk of early recurrence after definitive therapy [ 49, 50]. Several series have documented that most recurrences occur in the first 2 years after completion of treatment [51, 52]. Tabouret et al. demonstrated a switch in patients with Glioblastoma multiforme from VEGF pathway to CXCL12 /CXCR4 pathway [53–55]. Potentially, this mechanism could apply for esophageal cancer with early recurrence [56].

Taking again into account the small number of cases in this retrospective analysis, histopathological status seems to be no predictor for CXCR4 expression, in line with results of Kaifi et al. and Gockel et al. [14, 57] It is difficult to state beyond doubt that Pentixafor PET/CT results correlate with immunohistochemistry at *n* = 10. However, in patients #4 and #9, the IHC measured CXCR4 expression (strong respectively intermediate) was consistent with a coelic lymph node conglomeration (= metastasis) either an adrenal metastasis on the left side. Patient #4 during initial staging; patient #9 for re-staging after radiochemotherapy, which is why the results should at least be considered an exciting trend. For comparative assessment of FDG and Pentixafor uptake, the mediastinum may represent a suitable reference region. High tumor-uptake and low CXCR4 expression in non-tumor regions indicate promising preconditions for a CXCR4 specific radionuclide therapy.

Despite promising results, this pilot research work comes with limitations. First, only a limited number of patients could be included in the investigation. Secondly, both immunohistochemistry was not available in all cases and histological sampling was performed in three patients after nCRT. Third, it is a retrospective analysis, with inherent bias, although our institutional database is managed prospectively with strict tracking of all patients. As a consequence, a controlled study is now needed to shed more light on the potential diagnostic and therapeutic benefits of Pentixafor PET/CT in esophageal cancer.

## Conclusion

Our data shows that additional PET/CT imaging with Pentixafor for imaging the CXCR4 chemokine receptor is feasible but heterogeneous in both newly diagnosed and pretreated recurrent esophageal cancer. Therefore, preliminary results of this retrospective analysis imply that information should be considered complementary to CT, EUS and FDG-PET within three-modality (re-)staging. Of note, CXCR4 could provide an additional marker for metastatic tendency in adenocarcinoma or squamous cell carcinoma. In addition, the Pentixafor PET/CT may offer a diagnostic tool for radiation field expansion in radiooncology and thus possibly additional clinical benefit with regard to the oncological outcome.

## Supplementary Information

**Additional file 1: Supplementary Table 1.** SUVmean in background for FDG and Pentixafor.

## Data Availability

The datasets supporting the conclusions of this article are included within the article (and its additional files).

## Abbreviations

Adeno: Adenocarcinoma
CXCR4: Chemokine (C-X-C motif) Receptor 4
CT: Computerized Tomography
EARL: European Association of Nuclear Medicine and its Research Ltd.
ESCC: Esophageal (Squamous Cell) Cancer
EUS: Endoscopic Ultrasound
FDG: Fluorodeoxyglucose
IHC: Immunohistochemistry
MBP: Mediastinal Blood Pool
nCRT: Neoadjuvant Chemoradiotherapy
OGD: Oesophagogastroduodenoscopy
OSEM: Ordered Subset Expectation Maximization
PET/CT: Positron Emission Tomography/Computer Tomography
SC: Serum Concentrations
SCC: Squamous Cell Cancer
SUV: Standardized Uptake Value
UICC TNM: The Union for International Cancer Control Tumor Node Metastasis Classification of Malignant Tumours
