# Supplementary material for: Pentixafor PET/CT for imaging of chemokine receptor 4 expression in esophageal cancer – a first clinical approach
Source: Cancer Imaging. 2021 Feb 12;21:22. doi: 10.1186/s40644-021-00391-w (PMC7881561; doi:10.1186/s40644-021-00391-w)
Supplement: Supplementary file 1 — Additional file 1: Supplementary Table 1. SUVmean in background for FDG and Pentixafor. [file 40644_2021_391_MOESM1_ESM.docx]

**Supplementary Data**

| **Supplementary Table 1:** SUVmean in background for FDG and Pentixafor | | | | | | | | | | |
| --- | --- | --- | --- | --- | --- | --- | --- | --- | --- | --- |
| Patient no. | FDG SUVmean | | | | | Pentixafor SUVmean | | | | |
|  | Liver | Mediastinum | Spleen | Bone marrow | Brain | Liver | Mediastinum | Spleen | Bone marrow | Brain |
| #1 | 2,12 | 1,66 | 1,76 | 1,43 | 7,01 | 2,04 | 1,48 | 4,32 | 2,13 | 0,16 |
| #2 | 1,98 | 1,45 | 1,11 | 0,99 | 6,64 | 1,13 | 1,45 | 4,36 | 0,84 | 0,11 |
| #3 | 3,32 | 2,06 | 2,21 | 0,74 | 8,09 | 1,05 | 2,49 | 5,51 | 0,67 | 0,21 |
| #4 | 2,9 | 2,02 | 2,04 | 1,82 | 4,67 | 1,15 | 1,64 | 6,84 | 1,85 | 0,11 |
| #5 | 2,36 | 1,58 | 1,87 | 1,24 | 10,44 | 1,58 | 1,85 | 4,78 | 2,67 | 0,12 |
| #6 | 2,83 | 2,26 | 2,04 | 1,42 | 10,39 | 1,4 | 1,66 | 6,47 | 1,23 | 0,1 |
| #7 | 2,35 | 0,65 | 1,85 | 2,25 | 5,91 | 1,22 | 0,91 | 5,21 | 2,83 | 0,17 |
| #8 | 2,7 | 1,75 | 1,89 | 0,82 | 7,61 | 1,23 | 1,68 | 6,85 | 0,79 | 0,27 |
| #9 | 2,27 | 1,77 | 1,58 | 1,09 | n.a.* | 1,02 | 1,96 | 5,04 | 1,8 | 0,47 |
| #10 | 2,32 | 1,39 | 1,86 | 1,38 | 8,94 | 1,72 | 1,88 | 6,09 | 1,08 | 0,29 |
| **Mean** | 2.52 | 1.66 | 1.82 | 1.32 | 7.74 | 1.35 | 1.70 | 5.55 | 1.59 | 0.201 |
| **Median** | 2.36 | 1.71 | 1.87 | 1.31 | 7.61 | 1.23 | 1.67 | 5.36 | 1.52 | 0.165 |
| **Range** | 1.98 - 3.32 | 0.650 - 2.26 | 1.11 - 2.21 | 0.740 - 2.25 | 4.67 - 10.4 | 1.02 - 2.04 | 0.910 - 2.49 | 4.32 - 6.85 | 0.670 - 2.83 | 0.100 - 0.470 |
| **SD** | 0.411 | 0.449 | 0.303 | 0.459 | 1.95 | 0.331 | 0.407 | 0.966 | 0.785 | 0.116 |
| **IQR** | 0.515 | 0.475 | 0.220 | 0.413 | 2.30 | 0.400 | 0.353 | 1.53 | 1.16 | 0.143 |
| *IQR* interquartile range, *SD* standard deviation  right part of the liver, in the mediastinum, in the spleen, in the bone marrow and in the brain; *not available in 1 patient | | | | | | | | | | |
